# Supplementary material for: COZOID: contact zone identifier for visual analysis of protein-protein interactions
Source: BMC Bioinformatics. 2018 Apr 6;19:125. doi: 10.1186/s12859-018-2113-6 (PMC5889581; doi:10.1186/s12859-018-2113-6)
Supplement: Supplementary file 2 — Software build. Executable binary file of the software tool. (ZIP 118,784 kb) [file 12859_2018_2113_MOESM2_ESM.zip › cozoid/java/docs/beansbinding-1.2.1-doc.zip!/org/jdesktop/el/impl/lang/FunctionMapperFactory.html]

FunctionMapperFactory (Beans Binding)


---


|  |  |  |
| --- | --- | --- |
| **Overview** \| **Package** \| **Class** \| **Use** \| **Tree** \| **Deprecated** \| **Index** \| **Help** | |  |
| **PREV CLASS**   **NEXT CLASS** | **FRAMES**    **NO FRAMES**     **All Classes** |  |
| SUMMARY: NESTED \| FIELD \| CONSTR \| METHOD | DETAIL: FIELD \| CONSTR \| METHOD |  |


---


## org.jdesktop.el.impl.lang Class FunctionMapperFactory

```
java.lang.Object
  org.jdesktop.el.FunctionMapper
      org.jdesktop.el.impl.lang.FunctionMapperFactory
```

---

``` public class FunctionMapperFactory extends FunctionMapper ```

---

| **Field Summary** | |
| --- | --- |
| `protected  FunctionMapperImpl` | `memento` |
| `protected  FunctionMapper` | `target` |


**Constructor Summary**

`FunctionMapperFactory(FunctionMapper mapper)`


| **Method Summary** | |
| --- | --- |
| `FunctionMapper` | `create()` |
| `java.lang.reflect.Method` | `resolveFunction(java.lang.String prefix, java.lang.String localName)`             Resolves the specified prefix and local name into a `java.lang.Method`. |

**Methods inherited from class java.lang.Object**

`clone, equals, finalize, getClass, hashCode, notify, notifyAll, toString, wait, wait, wait`

**Field Detail**

### memento

```
protected FunctionMapperImpl memento
```

---


### target

```
protected FunctionMapper target
```


**Constructor Detail**

### FunctionMapperFactory

```
public FunctionMapperFactory(FunctionMapper mapper)
```


**Method Detail**

### resolveFunction

```
public java.lang.reflect.Method resolveFunction(java.lang.String prefix,
                                                java.lang.String localName)
```

:   **Description copied from class: `FunctionMapper`**
:   Resolves the specified prefix and local name into a
    `java.lang.Method`.

    Returns `null` if no function could be found that matches
    the given prefix and local name.

    :   **Specified by:**: `resolveFunction` in class `FunctionMapper`
    :   **Parameters:**: `prefix` - the prefix of the function, or "" if no prefix. For example, `"fn"` in `${fn:method()}`, or `""` in `${method()}`.: `localName` - the short name of the function. For example, `"method"` in `${fn:method()}`. **Returns:**: the static method to invoke, or `null` if no match was found.

---


### create

```
public FunctionMapper create()
```


---


|  |  |  |
| --- | --- | --- |
| **Overview** \| **Package** \| **Class** \| **Use** \| **Tree** \| **Deprecated** \| **Index** \| **Help** | |  |
| **PREV CLASS**   **NEXT CLASS** | **FRAMES**    **NO FRAMES**     **All Classes** |  |
| SUMMARY: NESTED \| FIELD \| CONSTR \| METHOD | DETAIL: FIELD \| CONSTR \| METHOD |  |


---
